# Direct Reprograming of Mouse Fibroblasts into Dermal Papilla Cells via Small Molecules

**DOI:** 10.3390/ijms23084213

**Published:** 2022-04-11

**Authors:** Yihe Ma, Yumiao Lin, Wenting Huang, Xusheng Wang

**Affiliations:** School of Pharmaceutical Sciences (Shenzhen), Sun Yat-sen University, Shenzhen 518107, China; mayh23@mail2.sysu.edu.cn (Y.M.); linym33@mail2.sysu.edu.cn (Y.L.); huangwt67@mail2.sysu.edu.cn (W.H.)

**Keywords:** direct reprogramming, fibroblasts, dermal papilla, small molecules

## Abstract

The reprogramming of somatic fibroblasts into alternative cell linages could provide a promising source of cells for regenerative medicine and cell therapy. However, the direct conversion of fibroblasts into other functional cell types is still challenging. In this study, we show that dermal-papilla-cell-like cells (DPC-LCs) can be generated by treating fibroblasts, including L929 mouse fibroblast cell lines and somatic mouse fibroblasts, with small molecules. Based on alkaline phosphatase activity and other molecular markers, different compounds or their combinations are needed for converting the two different fibroblasts into DPC-LCs. Notably, we found that TTNPB alone can efficiently convert primary adult mouse fibroblasts into DPC-LCs. DPC-LCs generated from mouse fibroblasts showed a stronger hair-inducing capacity. Transcriptome analysis reveals that expression of genes associated with a hair-inducing capacity are increased in DPC-LCs. This pharmacological approach to generating functional dermal papilla cells may have many important implications for hair follicle regeneration and hair loss therapy.

## 1. Introduction

Regenerative medicine aims to repair and regenerate injured tissues, thereby restoring their damaged function [1]. In 2006, Yamanaka successfully generated induced pluripotent stem cells (iPSCs) in mice through the viral transduction of four transcription factors into mouse somatic cells [2]. Subsequently, the same approach was used to induce human iPSCs in the following year [3]. This breakthrough in stem cell biology has sparked enthusiasm among researchers for therapeutic developments in tissue and organ repair or regeneration via cell-based therapy. However, generating iPSCs via viral transduction has the disadvantages of low recombination efficiency and safety risks, such as potential tumorigenicity and dorm genome changes due to viral integration [4]. Small molecules are being used to improve the efficiency of reprogramming and even to substitute for the use of transcription factors [5,6]. In 2013, significant progress was made in inducing mouse iPSCs using only small-molecule compounds [7]. Small molecule compounds replace transcription factors, providing a safer and more efficient pathway for the preparation of iPSCs. Since then, exciting results have been achieved in the induction and differentiation of pluripotent stem cells, somatic lineage transformation, and in vitro or in vivo regulation of adult stem cells using small-molecule chemical methods [8,9,10,11,12].

Hair loss is a common disease caused by various factors such as illness, age, and mental stress. It is a sub-health problem that affects billions of men and women around the world [13]. With the advances in tissue engineering and regenerative medicine, regenerating hair follicles through a cell-based approach provides an alternative therapy for hair loss. The hair follicle is a mini-organ that is composed of epithelial and dermal compartments, and the cross-talk between these two compartments plays an important role in the morphogenesis and growth of the hair follicle [14,15]. Dermal papilla (DP) cells are the main dermal compartments of the hair follicle, which are located at the base of the hair follicle [16]. The indispensable role of DP cells in hair follicle development and regeneration was already demonstrated decades ago [17,18,19,20,21,22]. However, cultured DP cells were reported to lose their hair-inducing activity quickly once they were removed from their hair follicle microenvironment [23], which makes it a big challenge to acquire enough hair-inducing DP cells for hair loss treatment.

In addition to DP cells, freshly isolated neonatal mouse dermal fibroblasts have also been demonstrated to have the capacity to induce HF neogenesis [24,25], but such HF inductivity of dermal fibroblasts is not shown in adults. Fan et al. conferred this hair-inducing capacity upon adult mouse fibroblasts by treating them with cell-free extract from embryonic skin [26]. In addition, DP cells and dermal fibroblasts were reported to originate from common fibroblast progenitors during mouse skin embryonic development, since both have highly homologous gene expression profiles [27]. Considering that the chemical transition of mouse fibroblasts directly to other cell types has been successfully implemented [28,29], its transformation into DP cells should be easier to achieve. Moreover, Zhao et al. found that human fibroblasts can be transformed into hair-inducing dermal papilla cells via treatment of FGF2, BMP2 and BIO [30].

Despite important progress having been achieved in transforming fibroblasts into DP cells, the induction efficiency and hair-induction capacity need to be improved. In this study, we try to establish a more efficient method for transforming fibroblasts into DP cells by chemical induction. In addition to regulating hair follicle development and growth, DP is thought to act as a reservoir of pluripotent stem cells. Small molecules used to replace transcription factors and increase reprogramming efficiency during the reprogramming of fibroblasts into iPSCs may also be helpful in promoting the transformation into DP cells. Moreover, we found that several chemical compounds, or their combinations, can effectively transform two different mouse fibroblasts into dermal-papilla-cell-like cells (DPC-LCs).

## 2. Results

### 2.1. Three-Compound Combination Efficiently Induces L929 Mouse Fibroblast Cell Lines to DPC-LCs

**Figure 1:**
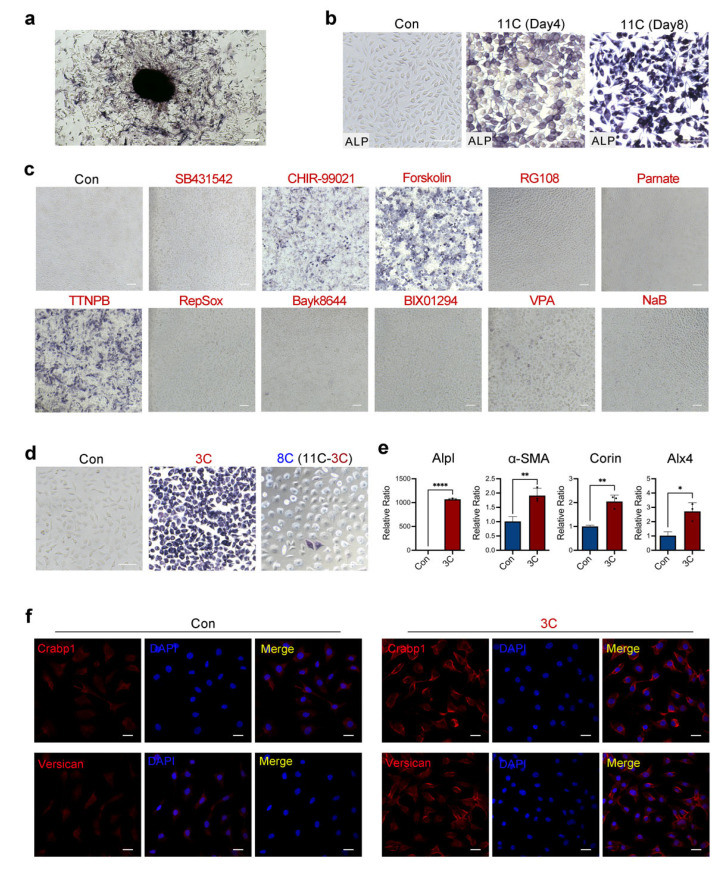
Three-compound combination efficiently induces L929 fibroblasts to dermal-papilla-cell-like cells (DPC-LCs). (**a**) Alkaline phosphatase (ALP) staining of mouse palpate dermal papilla cells. Scale bars, 200 μm. (**b**) ALP staining of L929 cells after induction with the combination of eleven small molecules (11C) on the 4th day and the 8th day, respectively. Scale bars, 100 μm. (**c**,**d**) ALP staining of L929 cells after induction with a single small molecule and other combinations on day 8. Scale bars, 100 μm. (**e**) Expression of key marker genes of Dermal papilla (DP) in control or 3C-treated L929 on 8th day was detected by quantitative real-time PCR (RT-PCR) (mean ± SD; *n* = 3). * *p* < 0.05; ** *p* < 0.01; **** *p* < 0.0001. (**f**) Immunofluorescence staining of DP markers after the induction of control or 3C-treated L929 on day 8. Scale bars, 20 μm.

To screen small molecules that can induce the transformation of mouse fibroblasts into DPC-LCs, the L929 mouse fibroblast cell line was first used in this study. We initially screened a panel of 11 candidate chemicals, including SB431542, CHIR99021, Forskolin, RG108, Parnate, TTNPB, RepSox, Bayk8644, BIX01294, VPA and NaB, which are known to facilitate reprogramming or functionally replace one or more of the four reprogramming transcription factors (Table 1) [7,31,32,33,34]. Alkaline phosphatase (ALP) as a useful marker of functional DP is associated with the trichogenicity of DP cells [35]. We confirmed through ALP staining that isolated dermal papilla cells showed strong ALP activity (Figure 1a). ALP staining is a simple and effective way to determine ALP activity; it was therefore used to screen for candidate chemicals that may induce the transformation. We first tested whether a combination of 11 candidate small molecules could efficiently induce L929 ALP activity. The cells were treated with 11 small-molecule cocktails (11C) and then ALP staining was performed every four days. Some of the cells were stained positively for ALP after 4 days of treatment with 11C, and all the cells showed strong positive staining for ALP on the 8th day. No positive ALP staining was detected in the control group, in which the cells were treated with DMSO (Figure 1b). To determine whether defined factors can induce ALP positivity in 11C, the L929 cells were treated with a single compound of the 11C for 8 days. Only three compounds significantly induced the ALP activity of L929, and these three compounds were CHIR99021, Forskolin and TTNPB (Figure 1c). Notably, the combination of these three compounds (3C) was sufficient to induce L929 cells to have 100% ALP positivity, while the combination of the eight remaining compounds showed only weak ALP positivity (Figure 1d). These results indicate that the combination of CHIR99021, Forskolin and TTNPB is sufficient to induce L929 cells to transform into DPC-LCs according to ALP activity.

Based on these results, we next investigated whether other well-defined DP cell markers, besides Alpl, were expressed in 3C-treated cells. In addition, quantitative real-time PCR (RT-PCR) combined with an immunofluorescence assay showed that the expression of other DP markers, including α-SMA, Corin, Alx4, Crabp1 and Vcan, was also significantly increased in 3C-treated L929 (Figure 1e,f). Furthermore, the expression level of *Alpl* in 3C-treated L929 cells was also verified by quantitative RT-PCR, and the *Alpl* expression level was significantly increased by more than one thousand times compared with the control group (Figure 1e). Collectively, these findings suggest that the three-compound cocktail can initiate DP fates in mouse fibroblasts.

### 2.2. TTNPB Induces Primary Mouse Fibroblast Conversion into DPC-LCs

**Figure 2:**
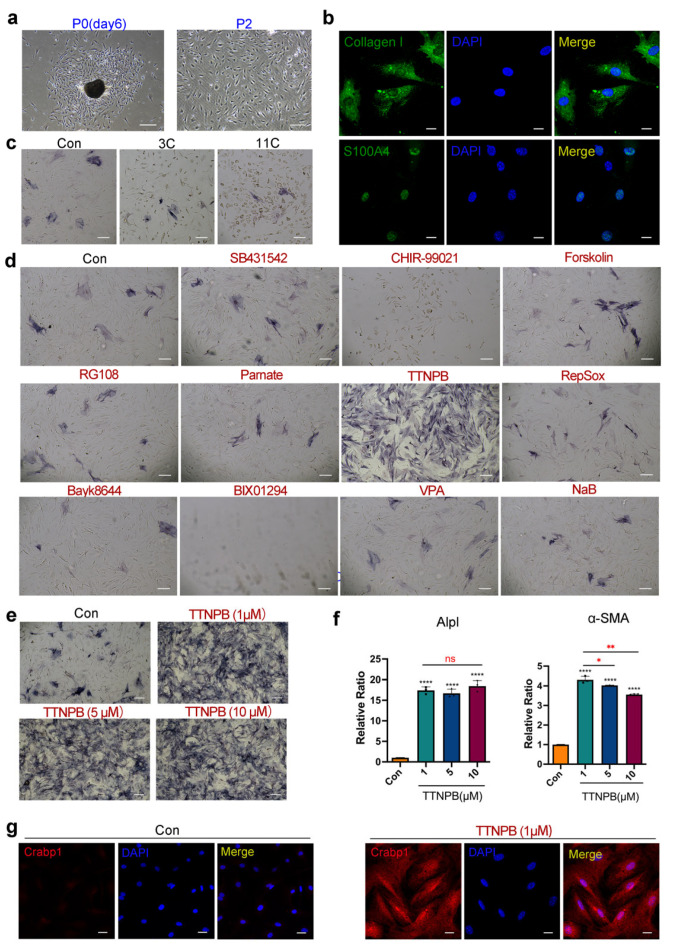
TTNPB induces primary adult mouse fibroblast conversion into DPC-LCs. (**a**) Morphology of primary fibroblasts extracted from the dorsal skin of adult mice. Scale bars, 200 μm. (**b**) Immunofluorescence staining of fibroblast markers in primary adult mouse fibroblasts. Scale bars, 20 μm. (**c**,**d**) ALP staining of primary adult mouse fibroblasts on day 8 after induction with a single small molecule or two combinations (3C and 11C). Scale bars, 200 μm. (**e**) ALP staining of primary adult mouse fibroblasts after induction with different concentrations of TTNPB on day 8. Scale bars, 200 μm. (**f**) Expression levels of *Alpl* or *α-SMA* in different concentrations of TTNPB-treated primary adult mouse fibroblasts on day 8 by quantitative RT-PCR (mean ± SD; *n* = 3). * *p* < 0.05; ** *p* < 0.01; **** *p* < 0.0001; ns, not significant (*p* > 0.05). (**g**) Immunofluorescence staining of Crabp1 in primary adult mouse fibroblasts that were treated with DMSO or TTNPB (1 μM) for 8 days. Scale bars, 20 μm.

Immortalized cell lines are most commonly used in biological experiments, especially in large-scale drug screening, because they are readily available and can be expanded without limitation. However, the behavior of cell lines may differ from what occurs in vivo in some important respects [36]. The importance of using primary cells, rather than immortalized cell lines, to perform biological experiments is becoming widely recognized. Based on the previous results in mouse fibroblast cell lines (L929), we further induced mouse primary cells with the above small molecules. Firstly, primary mouse fibroblasts were isolated from the dorsal skin of adult mice and expanded as described previously [37]. Within 2–5 days, the fibroblasts crawled out of the skin tissue fragments and became attached to the plate. Once the wells reached confluency, the fibroblasts were passaged for two more passages with Eagle’s minimum essential medium (EMEM) containing 15% FBS, 1% P/S, non-essential amino acids, and sodium pyruvate, which only support the growth of fibroblasts. The extracted cells had a typical fibroblast morphology with an elongated, spindle-shaped cell body that grows aligned and in bundles when confluent (Figure 2a). Moreover, immunofluorescence staining identified the cells as positive for fibroblast markers (Collagen I and S100A4) (Figure 2b).

Inconsistent with the results of the L929 cell line, neither the 3C nor the 11C combination enhanced ALP activity in primary mouse fibroblasts (Figure 2c). Moreover, only a single small-molecule treatment with TTNPB significantly increased ALP activity (Figure 2d). Therefore, we used the small-molecule TTNPB in further experiments. Firstly, the concentration of TTNPB was further optimized. ALP staining was performed in primary adult mouse fibroblasts after induction with different concentrations of TTNPB for 8 days. Interestingly, different concentrations of TTNPB (1, 5 and 10 μM) showed the same ALP-positive efficiency (Figure 2e). Assayed by quantitative RT-PCR (Figure 2f), there was also no significant difference in the gene expression level of *Alpl* among the treatment groups with different concentrations of TTNPB. Moreover, the expression level of *α-SMA* was significantly increased in all tested concentrations, with the highest expression at the concentration of 1 μM. Therefore, a concentration of 1 μM was selected for subsequent induction of TTNPB.

Crabp1 is expressed in dermal agglutinates as early as the embryonic development of hair follicles and is expressed in DP cells throughout the whole hair growth cycle, including in anagen and telogen follicles in the postnatal mice, but is not detected in dermal fibroblasts [5]. Crabp1 immunofluorescence staining showed no expression in the DMSO-treated mouse fibroblasts, which is consistent with the previous study of Crabp1 expression in fibroblasts in vivo. By contrast, immunofluorescence staining of Crabp1 showed strong expression in transformed cells after TTNPB treatment for 8 days (Figure 2g). Collectively, the results of ALP staining, quantitative RT-PCR and immunofluorescence staining strongly suggest that transformed mouse fibroblasts acquire some DP molecular feature. However, the hair-inducing capacity of DPC-LCs requires further examination.

### 2.3. DPC-LCs Converted from Mouse Fibroblasts by TTNPB Acquired Hair-Inducing Capacity

**Figure 3:**
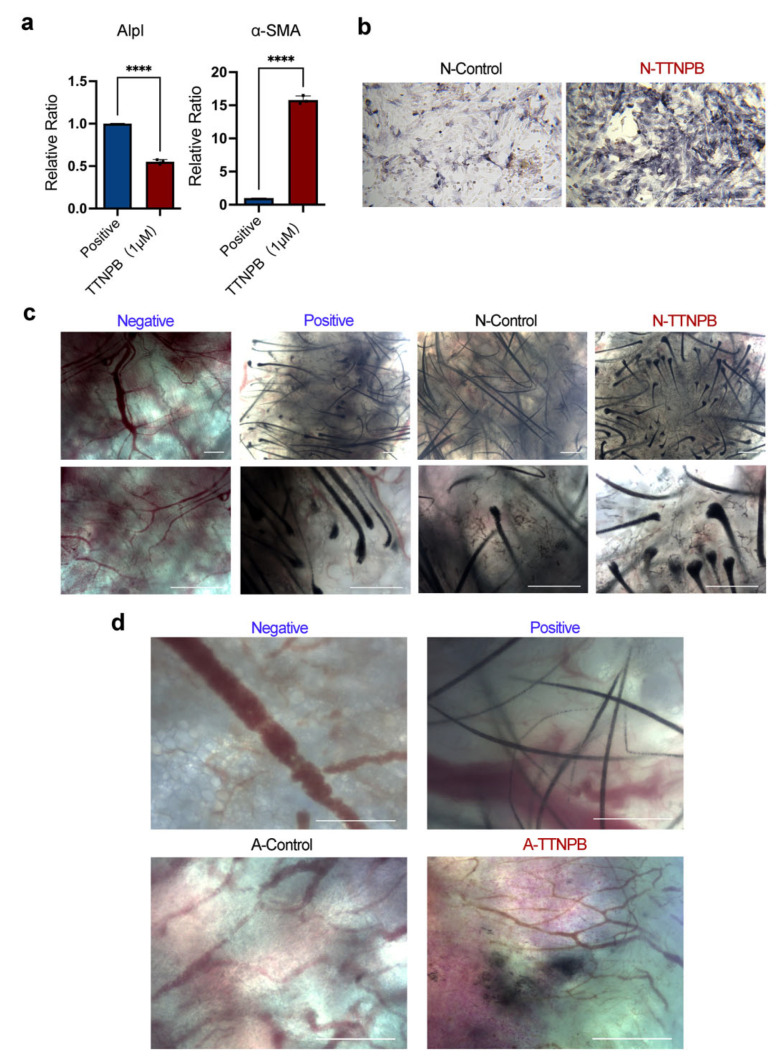
TTNPB enhances the hair-inducing capacity of neonatal mouse fibroblasts and confers a hair-forming ability on adult mouse fibroblasts. (**a**) Expression levels of *Alpl* or *α-SMA* in TTNPB-treated primary adult mouse fibroblasts on day 8 by quantitative RT-PCR (mean ± SD; *n* = 3), and neonatal mouse fibroblasts (P0) as positive. **** *p* < 0.0001. (**b**) ALP staining of neonatal mouse fibroblasts after 24 h treatment with TTNPB (1 μM). Scale bars, 100 μm. (**c**) Microscope picture of the hair follicle reconstitution assay, in which mouse epidermal cells were combined with neonatal mouse fibroblasts treated with TTNPB (1 μM) or DMSO. Scale bars, 200 μm. (**d**) Microscope images of nude mice skin whole mounts transplanted with newborn mouse epidermal cells combined with TTNPB- (1 μM) or DMSO-treated adult fibroblasts. Scale bars, 200 μm. Freshly isolated neonatal mouse fibroblasts were used as a positive control, and only keratinocytes were injected for the negative control in (**c**,**d**).

Before investigating the hair-inducing capacity of DPC-LCs, we first compared the gene expression of *Alpl* and *α-SMA* between neonatal mouse dermal fibroblasts and TTNPB-treated adult mouse fibroblasts. As shown in Figure 3a, the expression of *Alpl* in adult mouse fibroblasts induced by TTNPB was increased to half that of newborn mouse fibroblasts. Moreover, *a-SMA*, a marker of DP in vitro but not in vivo, was more expressed than neonatal murine fibroblasts, which was also consistent with the report. Next, we determined whether TTNPB could improve the ability of neonatal mouse fibroblasts to induce hair. The neonatal mouse dermal fibroblasts were treated with TTNPB or DMSO (denoted as the groups of N-TTNPB and N-control) for 24 h before ALP staining and in vivo hair follicle reconstitution. Notably, the ALP activity of neonatal mouse fibroblasts was also significantly enhanced by TTNPB (Figure 3b). Additionally, hair follicle reconstitution was performed as described [38] to evaluate the hair-inducing capacity of TTNPB- or DMSO-treated neonatal mouse fibroblasts. Freshly isolated neonatal mouse fibroblasts were used as a positive control, and only keratinocytes were injected for the negative control. Three weeks after implantation, we found HF formation in all implants with neonatal mouse fibroblasts, including freshly isolated (positive group) and cultured cells (TTNPB- or DMSO-treated), while no hair follicles were observed in the negative group. The hair follicles formed in the TTNPB-treated group showed a larger hair follicle bulb compared to the control group, indicating the enhanced hair-inducing capacity of TTNPB-treated fibroblasts (Figure 3c).

Wound-induced hair follicle neogenesis (WIHN) is an important model for studying hair follicle regeneration during wound repair [39]. Quan M. Phan et al. revealed that upper wound fibroblasts expressing Crabp1 play an important role in WIHN. This cell type also shares a similar gene signature with the murine papillary fibroblast lineage, which is indispensable for supporting hair follicle morphogenesis and growth [40]. Given the strong expression of Crabp1 in DPC-LCs, we further tested the HF inductivity of the DPC-LCs transformed from mouse fibroblasts in the full-thickness wound. Furthermore, the combination of keratinocytes and TTNPB-treated cells (denoted by the group of A-TTNPB) induced HF-like structure formation and pigmentation, whereas the group with DMSO-treated cells (denoted by the group of A-control) showed neither HF formation nor HF pigmentation (Figure 3d). The results further support the hypothesis that TTNPB can functionally transform fibroblasts into DPC-LCs, which not only have the ability to induce hair follicles in vivo but may also play a role in hair follicle pigmentation.

### 2.4. TTNPB Induced Dramatic Changes in Gene Expression Profile in Adult Mouse Fibroblasts

To further elucidate the mechanism that converts mouse fibroblasts into DPC-LCs by TTNPB, we performed RNA-sequencing for TTNPB-treated (treated) and DMSO-treated (control) adult mouse fibroblasts, with three samples from each group. The sequence data were uploaded into the Sequence Read Archive (SRA) at the National Centre for Biotechnology Information (NCBI) with an accession number of PRJNA817876.

An overview of the sequencing metrics and quality check results for RNA-sequencing raw reads is outlined in Table S1. After removing reads containing adapters, reads containing an N base and low-quality reads, the 43,066,674, 44,369,324 and 42,312,892 clean reads of the control groups (C1, C2 and C3) were obtained, and the 44,109,716, 42,842,164 and 45,132,988 clean reads were obtained from the TTNPB-treated groups (T1, T2 and T3). The sequencing error rate of each library was 0.03%, and more than ninety percent of the clean reads data had Phred-like quality scores at the Q30 level (an error probability of 0.001), indicating a high sequencing quality. The total mapping ratio of the reference genome varied from 94.64% to 95.72%, while small proportions (<5%) were mapped multiple loci of the reference genome (Table S2).

**Figure 4:**
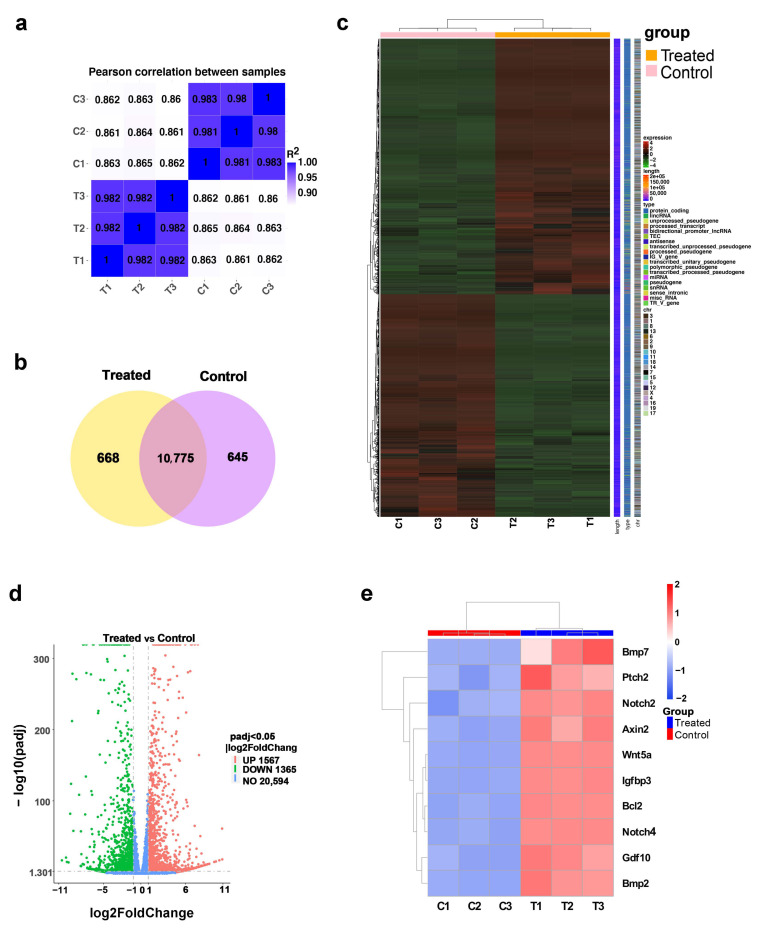
TTNPB caused significant changes in the gene expression profile of primary mouse fibroblasts. (**a**) Pearson’s correlation analysis between treated and control groups. (**b**) Venn diagram illustrating the number of shared genes between the two comparison groups (treated vs. control). (**c**) Gene expression heat map of treated and control groups. (**d**) The volcano plot depicts the quantity of differentially expressed genes (DEGs) and the specific distribution of DEGs in treated vs. control groups. (**e**) The heat map of DP signature genes expression in treated vs. control groups.

A Pearson’s correlation analysis was performed to examine the similarities and discrepancies between the two groups (Figure 4a). The results showed that samples in the same group were highly similar, while samples from different groups were distinct. A Venn diagram illustrates 10,775 genes shared between the two groups (Figure 4b). As shown in the cluster heat map, the reproducibility of the samples was further confirmed, and it was found that the treated and control groups were distinctly different in their global gene expression patterns (Figure 4c). A gene expression analysis on the transcripts was performed to further characterize differentially expressed genes (DEGs) in the treated vs. control group. A total of 2930 DEGs were identified, among which 1567 were upregulated and 1365 were downregulated in the TTNPB-treated group (Figure 4d). The heat map showed that the gene expression of other DP signature markers was also remarkably elevated by TTNPB (Figure 4e).

**Figure 5:**
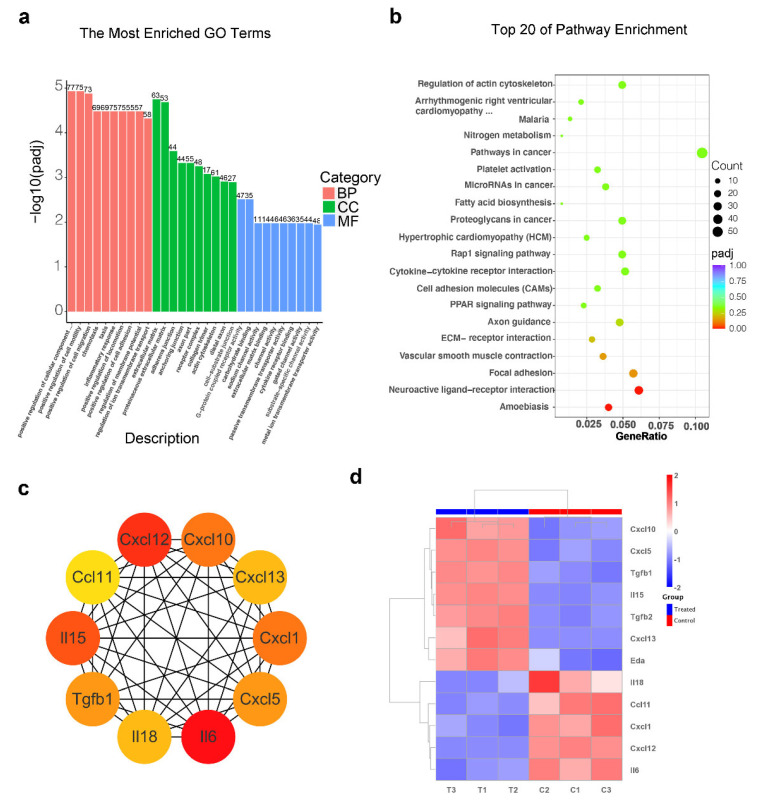
Gene Ontology (GO) and Kyoto Encyclopedia of Genes and Genomes (KEGG) analysis of differentially expressed genes by TTNPB treatment. (**a**) Analysis of Gene Ontology term enrichment in mouse fibroblasts with and without TTNPB treatment. (**b**) KEGG analysis of the top 20 upregulated pathways in the treated group compared to the control group. The size of the bubble represents gene number, color depth represents the padj, and the rich ratio represents the gene number/total gene number on the y-axis. (**c**) The PPI network of hub genes was analyzed by the degree method, with 10 nodes and 44 edges. The color depth of the nodes represents the degree level; the deeper the color, the higher the degree. (**d**) Heat map of cytokine expression in treated vs. control groups.

Gene Ontology (GO) functional annotation and Kyoto Encyclopedia of Genes and Genomes (KEGG) pathway analysis were also performed. In GO annotation, the most enriched processes in the treated group included the positive regulation of the component (BP category), extracellular matrix (CC category) and G-protein-coupled receptor activity (MF category) compared with the control group (Figure 5a). KEGG analysis of the up-regulated DEGs in treated vs. control group showed the top 20 enriched pathways, including platelet activation, the Rap1 signaling pathway, cytokine–cytokine receptor interaction, cell adhesion molecules (CAMs), the PPAR signaling pathway, ECM–receptor interaction, vascular smooth muscle contraction and neuroactive ligand–receptor interaction (Figure 5b).

An increasing amount of research suggests an interaction between chemokines and skin appendage morphogenesis [41,42,43]. Moreover, the result of the cytokine–cytokine receptor interaction pathway was also enriched in the TTNPB-treated group. To investigate the expression of cytokines after TTNPB treatment, a protein–protein interaction (PPI) network was built to explore the hub genes in the cytokine–cytokine receptor interaction pathway using Cytoscape. The top 10 hub genes, which were the genes exhibiting the most significant interaction, were identified using the Cytoscape CytoHubba application according to 12 topological algorithms (Table S3). The hub genes of at least five algorithms that overlapped were obtained, containing ten hub genes (*Il6*, *Cxcl12*, *Il15*, *Tgfb1*, *Cxcl1*, *Il18*, *Ccl11*, *Cxcl10*, *Cxcl13* and *Cxcl5*) (Figure 5c). The expression of *Cxcl10*, *Cxcl5*, *Tgfb1*, *Il15* and *Cxcl13* was upregulated in the TTNPB-treated group, while *Il18*, *Ccl11*, *Cxcl1*, *Cxcl12* and *Il6* were downregulated (Figure 5d). Notably, *Il6* was identified as the top hub gene among eight algorithms. It is reported that Il6 mRNA and proteins are upregulated in balding DP cells in male-pattern baldness, and dihydrotestosterone-inducible Il6 inhibits the elongation of human hair shafts and promotes the regression of hair follicles in mice [43]. In addition, some cytokines that have been reported to affect hair growth were also differentially expressed in the TTNPB-treated group (Figure 5d). Tgf-β2 was specifically expressed in hDPCs at higher levels compared to hDFs. Inhibition of the Tgf-β2 signal at either the ligand or the receptor level impaired hair folliculogenesis [44]. The EDA pathway is one of the most important evolutionary conserved pathways for regulating the morphogenesis of skin appendages, and its role in hair follicle biology has been extensively studied [45]. Lefebvre et al. identificated two chemokines, *cxcl10* and *cxcl11*, as primary hair placode-specific transcriptional targets of Eda [42]. Based on this result, the expression of these chemokines under TTNPB treatment may be involved in the changes in cell transformation. These bioinformatics data suggest that these signal pathways might be involved in the transformation of fibroblasts into DPC-LCs, while the underlying mechanism of the transformation of fibroblasts to DPC-LCs still needs further systematic analysis in future studies.

## 3. Discussion

In this study, we showed that DPC-LCs could be converted from two different mouse fibroblasts through chemical compounds. Based on their hair-inducing ability, DP cells are considered a promising cell source for cell replacement therapy for hair loss. Cell-based therapy involves reprogramming somatic fibroblasts or adult stem cells into alternative cell linages to arm them with enhanced or new functions, and finally enables acquiring the desired cell population for regenerative medicine [4]. iPSCs have characteristics similar to those of embryonic stem cells in terms of morphology, self-renewal and differentiation ability, while avoiding ethical issues [46]. Much effort has been devoted to obtaining DP cells from stem cells [47,48,49,50,51]. These processes are usually induced in multiple stages, which are time-consuming and quite expensive. Alternatively, the lineage conversion of cells enables the direct transition of one cell type to another, thus bypassing the pluripotent state, which is often faster in generating the target cell types.

Although cell-free extract from embryonic skin has been reported to induce HF neogenesis in adult mouse fibroblasts [26], the hair-forming ability was lost after further culturing in extract-free conditions, indicating that the obtained inductive state is not permanent. With this method, adult fibroblasts are only stimulated to the state necessary for reactivating HF morphogenesis, rather than being actually transformed into DP cells. Directly converting mouse fibroblasts into DP cells is still challenging.

The chemical composition of small molecules is clear and of high purity, and the difference between the batches is small. These molecules also have the characteristics of fast and dose-dependent biological activity. Appropriately targeted delivery and controlled release of small molecules can spatially and temporally modulate their in vivo actions [4]. Small molecules have been widely used in stem cell biology because of their unique advantages and have great potential in regenerative medicine. They can affect changes in cell fate by regulating multiple cellular processes, including the activities of histone deacetylase, histone demethylase and DNA methyltransferase, resulting in changes in cell transcription level, DNA epigenetics and histone modification, as well as in the Wnt pathway and TGF-B pathway. Eleven commonly used reprogramming small molecules involved in these cellular processes were selected for this study (Table 1). Here, we demonstrated a simple and feasible way to acquire DPC-LCs from mouse fibroblasts through chemical compounds.

Three compound cocktails containing CHIR99021, TTNPB and Forskolin can induce L929 cells to transform into DPC-LCs. By contrast, primary adult mouse fibroblasts can transform into DPC-LCs with TTNPB alone. These DPC-LCs, induced from two different types of mouse fibroblasts, acquired DP cell features at the molecular level. CHIR99021 is a glycogen synthase kinase three beta (GSK3) inhibitor that has been reported to activate Wnt/beta-catenin signaling without serious cell toxicities [52]. The Wnt pathway is considered to be the master regulator during hair follicle embryonic morphogenesis, and it is also critical for the postnatal neogenesis of HFs [53,54]. In addition, CHIR99021 has been reported to enhance the expression of DP signature genes associated with a hair-inducing ability in human DP cells [55]. Forskolin is an adenylate cyclase activator. It has been reported to promote hair epithelial cell growth and enhance the hair growth-promoting effect of procyanidin B-2 [56]. TTNPB is an analog of retinoic acid (RA) that potently and selectively activates retinoic acid receptors. RA is essential for the normal regulation of a variety of biological processes, including development, differentiation, proliferation and apoptosis [57,58,59]. RA has been shown to promote and regulate epithelial cell proliferation and differentiation, as well as vascular proliferation [60,61]. These factors are also important for promoting hair growth. Topical application of all-trans RA and other retinoids has been shown to produce minoxidil-like effects in C3H mouse models, namely, a prolonged growth phase and a shortened telogen period [62]. In addition, topical all-trans RA alone or in combination with 0.5% minoxidil has been shown to promote hair growth in patients with androgenetic alopecia [63,64]. The role of these small molecules in these signaling pathways is perhaps to promote the transformation of fibroblasts into DPC-LCs. However, it is not clear why CHIR99021 and FSK cannot induce the positive staining of ALP in primary adult mouse fibroblasts. The discrepancy in compounds between L929 cells and primary cells suggest that there are substantial differences between cell lines and primary cells, thus reducing the value of using cell lines to screen for reprogramming compounds in primary cells.

Hair-induction ability is also one of the most important features of functional DP cells. In this study, we tested the hair-induction ability of adult mouse fibroblasts transformed into DPC-LCs through an in vivo hair follicle recombination experiment. Neonatal mouse keratinocytes plus DMSO-treated adult mouse fibroblasts did not induce hair growth at the recipient site. Notably, in the subcutaneous tissue of nude mice implanted with DPC-LC combined with neonatal mouse keratinocytes, remarkable hair-follicle-like structures and pigmentation were observed. Together, in vivo and in vitro experiments demonstrate that TTNPB induces the transformation of mouse fibroblasts into DPC-LCs, which not only have DP-like molecular characteristics, but also have partial hair-induction ability. Although the DPC-LCs we induced successfully induced hair-follicle-like structures, they failed to induce hair with a complete structure, indicating that there is still a certain gap between the DPC-LCs and real DP cells. In our study, we only picked out 11 small molecules that might be useful. Although TTNPB-treated primary adult fibroblasts showed some characteristics of DP cells, the limited number of small molecules may have limited the efficiency of the transformation. To improve the efficiency of induction, experiments should be carried out with a greater number of small molecules in future studies.

In order to further understand the specific mechanism of TTNPB-induced mouse fibroblast transformation into DPC-LCs, a transcriptome sequencing analysis was performed. By analyzing the DEGs between the treated and control groups, the upregulated enrichment pathway was revealed after TTNPB treatment. Platelets are the first cell types to reach the site of tissue injury and are actively involved in the healing process through a variety of mechanisms, including cell membrane adhesion, aggregation, clot formation and the release of substances that promote tissue repair and regeneration [65,66]. Numerous studies have shown that platelet-rich plasma (PRP) can promote the healing of complex wounds in elderly patients [67]. PRP can promote hair regrowth in mice and AGA patients by inducing HFSC activation and proliferation, which leads to hair follicle regeneration [68,69,70]. Many studies have demonstrated the importance of cell adhesion molecules (CAMs) and the extracellular matrix (ECM) in skin development, and increasing evidence also shows that the ECM is important for HF progenitor cell fate determination throughout HF development [71,72,73]. Therefore, the upregulated enrichment of these pathways in the treated vs. control groups may be the key factors in inducing the transformation of fibroblasts into DPC-LCs. Hair follicle morphology relies on signaling pathways such as Wnt, Shh, Notch and BMP for its interactions with epithelial cells and mesenchymal cells. The Wnt pathway plays an important role in the induction of hair follicles, with Shh involved in morphogenesis and differentiation at late stages. Moreover, Notch signaling determines stem cell fate, and BMP is involved in cell differentiation [74]. Although some DP marker genes in these pathways were upregulated in treatment, these signaling pathways did not appear in the top 20 upregulation enrichment pathways, indicating that TTNPB does not act on these pathways to a large extent, which may be the cause of the inability to form a complete hair follicle structure. These questions will be further explored in future studies.

## 4. Materials and Methods

### 4.1. Animals

All the animal experiments were approved by the Institutional Animal Care and Use Committee of Sun Yat-sen University (Guangzhou, China). The animal study was approved by the Institutional Animal Care and Use Committee of Sun Yat-sen University (approval no. SYSU-YXYSZ-20210332). C57BL/6 mice and nude mice (BALB/c-nu/nu) were purchased from the Guangdong Medical Laboratory animal center (Guangdong, China). C57BL/6 mice 7–8 weeks old were used to isolate primary adult mouse fibroblasts, and 1–3-day-old mice were used to isolate neonatal mouse fibroblasts and epidermal cells. For invasive experiments, the animals were anesthetized with 1% (*v*/*v*) sodium pentobarbital.

### 4.2. Cells Isolation and Culture

The L929 cells (an areolar-derived fibroblast cell line) were purchased from Zhong Qiao Xin Zhou Biotechnology Co., Ltd. (Shanghai, China). The cells were cultured in MEM medium (Gibco, Grand Island, NY, USA) containing 10% fetal bovine serum (FBS; Gibco, Grand Island, NY, USA) and 1% penicillin/streptomycin (P/S, 100 U/mL; Gibco, Grand Island, NY, USA) and incubated at 37 °C in a 5% CO_2_ atmosphere.

Primary adult fibroblasts were isolated from the dorsal skin of 7-8-week-old C57BL/6 mice (the resting phase (telogen) of hair growth) as previously described [37]. Briefly, the dorsal skin was minced and incubated in DMEM/ F-12 medium with Liberase Blendzyme 3 (0.13 U/mL; Roche, Basel, Switzerland) and 1% P/S at 37 °C for 40 min. After washing with DPBS (Gibco, Grand Island, NY, USA) twice, the dissociated cells were plated in cell culture dishes with a complete DMEM/F-12 (Gibco, Grand Island, NY, USA) medium supplemented with 15% FBS and 1% P/S and were incubated at 37 °C in a 5% CO_2_ atmosphere. The cells were split and replated in Eagle’s minimum essential medium (EMEM; ATCC, Manassas, VA, USA) supplemented with 15% FBS and 1% P/S when reaching confluence. They were purified by further passaging in the EMEM medium.

Neonatal mouse fibroblasts and keratinocyte cells were obtained from newborn C57BL/6 mice as previously described [75]. The back skin of C57BL/6 mice was isolated and washed twice with PBS, and then incubated in 0.25% Trypsin without EDTA solution (Gibco, Grand Island, NY, USA) overnight at 4 °C. The next day, epidermal layers and dermal layers of the skin were isolated, separately homogenized with scissors and digested at 37 °C by collagenase I (Sigma, St. Louis, MO, USA) for 30 min at a concentration of 0.035% or 0.35%, respectively. The cell suspensions were passed through a cell strainer and washed in PBS. Moreover, the cells were used the same day or kept frozen for future use.

DPs were dissected from the vibrissae follicles of adult C57BL6 mice as previously reported [76]. The mystacial pad was cut open, the skin was inverted and the follicles were removed with fine forceps. The collagen capsules surrounding the vibrissae follicles were removed to expose the follicle end bulbs, and DPs were dissected using thin needles. Isolated DPs were placed on the bottom of cell culture dishes. The cell cultures were maintained in DMEM medium containing 10% FBS.

### 4.3. Small-Molecule Compounds

The small-molecule compounds used in our study were purchased from Selleck (Houston, TX, USA). The concentration of each compound is shown in Table 1.

### 4.4. Chemical Induction of DPC-LCs from Mice Fibroblasts

The mouse fibroblast cells (L929, adult mouse fibroblasts or neonatal mouse fibroblasts) were seeded in the culture plates. On the next day (day 0), the original medium was replaced with chemical reprogramming medium (DMEM supplemented with 10% FBS) containing the small-molecule compounds. The small-molecule-cocktail-containing medium was changed every 2 days. After 8 days, the DPC-LCs were harvested for further detection.

### 4.5. ALP Staining

The cells were fixed with 4% paraformaldehyde for 10 min at room temperature and washed three times with PBS. The fixed cells were stained with a BCIP/NBT Alkaline Phosphatase Color Development Kit (Beyotime, Shanghai, China) according to the manufacturer’s specification. Finally, the produced blue insoluble NBT formazan was observed under a light microscope.

### 4.6. Immunofluorescence-Staining Analysis

A total of 4% paraformaldehyde was used to fix the cells for 10 min, and then the cells were blocked with QuickBlockTM Blocking Buffer (Beyotime, Shanghai, China) containing 0.4% Triton X-100 for 1 h. The cells were incubated overnight at 4 °C with primary antibodies Crabp1 (Invitrogen, Carlsbad, CA, USA), Versican (Invitrogen, Carlsbad, CA, USA), Collagen I (Abcam, Bristol, UK) and S100A4 (Abcam, Bristol, UK). Then, the cells were allowed to interact with goat anti-mouse or anti-rabbit IgG H&L pre-adsorbed secondary antibodies (Abcam, Bristol, UK) for 1 h. Nuclear DNA was labeled in blue with DAPI. Finally, the image was taken with a confocal microscope (Leica, LSN880, Wetzlar, Germany).

### 4.7. Quantitative RT-PCR

Total RNA was extracted using the TRIzol regent (Invitrogen, Carlsbad, CA, USA) according to the manufacturer’s specification. A total of 1 μg of RNA was reverse transcribed into cDNA using the PrimeScript™ RT reagent Kit with a gDNA Eraser (Takara, Kusatsu, Japan), followed by RT-PCR with TB Green^®^ Premix Ex Taq™ II (Tli RNaseH Plus; Takara, Kusatsu, Japan), according to the manufacturer’s protocol. The primer sequences for the mouse genes were as follows: GAPDH (5′-AGAACATCATCCCTGCATCC-3′ and 5′-TCCACCACCCTGTTGCTGTA-3′), ALPL (5′-CAGGTCCCACAAGCCCGCAA-3′ and 5′-CCCGGTGGTGGGCCACAAAA-3′), a-SMA (5′-GTCCCAGACATCAGGGAGTAA-3′ and 5′-TCGGATACTTCAGCGTCAGGA-3′), Alx4 (5′-AACTACGCCCAGATTCAGAACC-3′ and 5′-GAGGGGACATACAGGCTGGT-3′), Corin (5′-ACATCCGGTATTGCCATTTGCCTCA-3′ and 5′-TCCCATAAAGTGGCCCAGTGCTT-3′). The relative level of gene expression was detected using the 2^−ΔΔCT^ method with glyceraldehyde-3-phosphate dehydrogenase (GAPDH) serving as a reference gene.

### 4.8. In Vivo HF Regeneration

HF neogenesis was tested in patch assays and wound-induced hair follicle neogenesis in 4–6-week-old female nude mice [38,77]. For the patch assays, 1 × 10^6^ cultured neonatal mouse fibroblast cells after treatment with TTNPB or DMSO were mixed with 1 × 10^6^ neonatal mouse keratinocytes in 100 μL PBS and injected into the hypodermis of nude mice with an 18-G needle. For the positive control, freshly isolated neonatal mouse fibroblasts were substituted for cultured neonatal mouse fibroblasts. Moreover, for the negative control, only keratinocytes were injected. The mice were sacrificed 3 weeks later, and the skin was removed for photography.

For wound-induced hair follicle neogenesis, two symmetrical full-thickness skin wounds with diameters of 4 mm were created on the back skin of nude mice by a skin biopsy punch. TTNPB- or DMSO-treated adult mouse fibroblasts (1 × 10^6^) were encased with neonatal mouse fibroblasts (1 × 10^6^) in 30 μL Matrigel (Corning, Corning, NY, USA). After incubation for 30 min at 37 °C, the cells–Matrigel mixture was implanted into the excisional wound. The wound was then covered with Tegaderm (3M). The mice were sacrificed 3–4 weeks later, and the skin was removed for photography.

### 4.9. RNA-Sequencing and Analysis

Adult mouse fibroblast cells treated with TTNPB or DMSO (Sigma, St. Louis, MO, USA) for 8 days were collected and the total RNA was extracted using the TRIzol regent. RNA purity and integrity were checked with the NanoPhotometer^®^ spectrophotometer (IMPLEN, Santa Clara, CA, USA) and the RNA Nano 6000 Assay Kit of the Bioanalyzer 2100 system (Agilent Technologies, Santa Clara, CA, USA), respectively. A total of 1 µg of RNA per sample was used for transcriptome sequencing. To generate sequencing libraries, the NEBNext^®^ UltraTM RNA Library Prep Kit for Illumina^®^ (NEB, Ipswich, MA, USA) was used, and index codes were added to attribute sequences to each sample. The clustering of the index-coded samples was performed on a cBot Cluster Generation System using the TruSeq PE Cluster Kit v3-cBot-HS (Illumina). After cluster generation, the library preparations were sequenced on an Illumina Novaseq platform, and 150 bp paired-end reads were generated.

For further analysis, clean data (clean reads) were obtained by removing reads containing adapters, reads containing ploy-*N* and low-quality reads from raw data. All the downstream analyses were based on the clean data with a high quality. Hisat2 v2.0.5 was used to build an index of the reference genome, and paired-end clean reads were aligned to the reference genome. FeatureCounts v1.5.0-p3 was used to count the read numbers mapped to each gene. Then, the FPKM of each gene was calculated based on the length of the gene and the reads count mapped to this gene. Differential expression analysis of two groups was performed using the DESeq2 R package (1.16.1). The resulting *p*-values were adjusted using Benjamini and Hochberg’s approach to controlling the false discovery rate (FDR). FDR <= 0.05 and |log2(foldchange)| ≥ 1 were set as the threshold for significantly differential expression. Gene Ontology (GO) enrichment analysis of differentially expressed genes was implemented by the cluster Profiler R package. GO terms with a corrected *p*-value of less than 0.05 were considered significantly enriched by differentially expressed genes. Moreover, the statistical enrichment of differentially expressed genes in KEGG pathways was also tested by the cluster Profiler R package. A protein–protein interaction (PPI) network was constructed using the STRING database. A plugin in the Cytoscape software, CytoHubba, was utilized to identify the top 10 hub genes using 12 different algorithms, including Degree, MCC, DMNC, MNC, BottleNeck, EcCentricit, Closeness, Radiality, Betweenness, Stress, Clustering Coefficient and EPC centrality analysis. The overlapping of 12 topological algorithms was carried out using the “UpSetR” package.

### 4.10. Statistical Analysis

The data were expressed as the mean ± standard deviation of the mean (S.D.). Comparisons between groups were performed with an unpaired *t*-test using the GraphPad Prism version 9.0 (GraphPad) software. Differentials were considered statistically significant at *p* < 0.05.

## 5. Conclusions

In summary, we showed that DPC-LCs can be generated from mouse fibroblast cell lines and primary adult mouse fibroblasts with a chemical compound cocktail (3C) or TTNPB, respectively. Although further investigation remains to be carried out, our current findings lay a foundation for converting the fibroblasts of mice into functional DPCs and provide an insight into the application of this chemical induction strategy on human cells, which has eventual implications for androgenic alopecia therapy.

## Figures and Tables

**Table 1:** Small-molecule compounds used in DPC-LC reprogramming of mouse fibroblasts.

No.	Full Name	Function	Concentration (μM)
1	SB431542	TGFβ inhibitor	2
2	CHIR99021	GSK3 inhibition	10
3	Forskolin	PKA activation	10
4	RG108	DNA methyltransferase inhibition	10
5	Tranylcypromine/Parnate	LSD1 histone demethylase inhibition	2
6	TTNPB	Retinoic acid receptor agonist	1
7	RepSox	TGFβ inhibitor	10
8	BayK8644	l-type Ca^2+^ channel activation	2
9	BIX01294	Histone methyltransferase inhibition	1
10	Valproic acid (VPA)	Histone deacetylase inhibition	500
11	Sodium butyrate (NaB)	Histone deacetylase inhibition	100

## Data Availability

The data presented in this study are available upon request from the corresponding author.

## Supplementary Materials

The following supporting information can be downloaded at: https://www.mdpi.com/article/10.3390/ijms23084213/s1.
