# Supplementary material for: Direct Reprograming of Mouse Fibroblasts into Dermal Papilla Cells via Small Molecules
Source: Int J Mol Sci. 2022 Apr 11;23(8):4213. doi: 10.3390/ijms23084213 (PMC9030401; doi:10.3390/ijms23084213)
Supplement: Supplementary file 1 [file ijms-23-04213-s001.zip › ijms-1658670-supplementary.pdf]

Supplementary Material

Table S1. Sequencing metrics and quality check results for RNA sequencing raw reads.

Sample name	Raw reads	Clean reads	Clean bases	Error rate	Q20 (%)	Q30 (%)	GC %
T1	45,945,762	44,109,716	6.62G	0.03	97.68	93.74	51
T2	44,778,854	42,842,164	6.43G	0.03	97.76	93.95	51.05
T3	47,303,014	45,132,988	6.77G	0.03	96.45	90.94	51.24
C1	44,941,598	43,066,674	6.46G	0.03	97.71	93.81	50.95
C2	46,414,448	44,369,324	6.66G	0.03	97.71	93.81	51.84
C3	44,171,190	42,312,892	6.35G	0.03	97.74	93.84	50.81

Q20 (%): the percentage of reads above quality score 20; Q30 %: the percentage of reads above quality score 30; GC %: the percentage of proportion of guanidine and cytosine nucleotides among total nucleotides.

Table S2. Alignment of sample sequencing data with reference genomes.

Sample name	Total Reads	Total Map	Multi Map	Read1 Map	Read2 Map
T1	44,109,716	42,114,225(95.48%)	1,877,921(4.26%)	20,156,595(45.7%)	20,079,709(45.52%)
T2	42,842,164	40,881,175(95.42%)	1,848,732(4.32%)	19,549,856(45.63%)	19,482,587(45.48%)
T3	45,132,988	42,713,461(94.64%)	1,884,225(4.17%)	20,583,056(45.61%)	20,246,180(44.86%)
C1	43,066,674	41,219,123(95.71%)	1,923,547(4.47%)	19,682,351(45.7%)	19,613,225(45.54%)
C2	44,369,324	42,312,157(95.36%)	1,823,724(4.11%)	20,278,454(45.7%)	20,209,979(45.55%)
C3	42,312,892	40,501,347(95.72%)	1,892,616(4.47%)	19,341,391(45.71%)	19,267,340(45.54%)

Table S3. Top 10 hub genes in network ranked by different methods.

Rank	Method											
	Degree	MCC	DMNC	MNC	BottleNeck	EcCentricity	Closeness	Radiality	Betweenness	Stress	Clustering-Coefficient	EPC
1	Il6	Cxcl5	Ccr10	Il6	Il6	Tnfsf11	Il6	Il6	Il6	Il6	Inhba	Il6
2	Cxcl12	Cxcl10	Ccl9	Cxcl12	Bmp4	Il6	Cxcl12	Cxcl12	Tgfb1	Tgfb1	Gdf7	Cxcl12
3	Il15	Cxcl13	Il16	Il15	Il15	Ngf	Il15	Tgfb1	Cxcl12	Cxcl12	Ackr3	Cxcl10
4	Cxcl1	Cxcl1	Ppbp	Cxcl1	Cxcl12	Tnfrsf12a	Tgfb1	Il15	Tnfrsf12a	Acvr2a	Il16	Cxcl1
5	Cxcl10	Ccl11	Cxcl14	Cxcl10	Il1rn	Cxcl12	Cxcl10	Tnfsf11	Il15	Bmp4	Gdf6	Il18
6	Cxcl5	Cxcl12	Ccl6	Cxcl5	Tnfrsf12a	Fas	Cxcl1	Cxcl10	Bmp4	Acvrl1	Gdf10	Cxcl5
7	Tgfb1	Il6	Ccl8	Cxcl13	Acvr2b	Tnfsf13b	Cxcl5	Cxcl1	Tnfsf11	Bmpr1b	Ccr10	Tgfb1
8	Cxcl13	Il15	Ackr3	Il18	Cxcl1	Clcf1	Il18	Cxcl5	Il18	Acvr2b	Ppbp	Il15
9	Il18	Il18	Inhba	Tgfb1	Tgfb1	Il11ra1	Tnfsf11	Il18	Ngfr	Bmp7	Il34	Cxcl13
10	Ccl11	Ccl9	Ccl11	Ccl11	Tnfsf13b	Ctf1	Cxcl13	Ccl11	Bmp7	Tnfrsf19	Il1rl2	Ccl11
